# Lateral Inhibition in the Vertebrate Retina: The Case of the Missing Neurotransmitter

**DOI:** 10.1371/journal.pbio.1002322

**Published:** 2015-12-10

**Authors:** Richard H. Kramer, Christopher M. Davenport

**Affiliations:** Department of Molecular and Cell Biology, University of California at Berkeley, Berkeley, California, United States of America

## Abstract

Lateral inhibition at the first synapse in the retina is important for visual perception, enhancing image contrast, color discrimination, and light adaptation. Despite decades of research, the feedback signal from horizontal cells to photoreceptors that generates lateral inhibition remains uncertain. GABA, protons, or an ephaptic mechanism have all been suggested as the primary mediator of feedback. However, the complexity of the reciprocal cone to horizontal cell synapse has left the identity of the feedback signal an unsolved mystery.

## Abstract 2

Our impressive sense of sight depends on negative feedback from horizontal cells to photoreceptors at the first synapse in the visual system, but the actual signal responsible for mediating this feedback remains mysterious. This article investigates the suspects.

## Introduction

It was a dark and stormy night. The lab seemed deserted, but shadowy figures could be made out in the dimly lit room where the two scientists worked. A flash of bright light suddenly shone on a small spot on the retina. The beam on the oscilloscope jumped, signaling a neural response. But here was the strange thing: when the scientists made the spot larger to illuminate more of the retina, the neural response got smaller, not bigger. A mysterious signal from the surrounding region was projecting laterally to inhibit the response in the center—but what was the signal?

**Fig 1:**
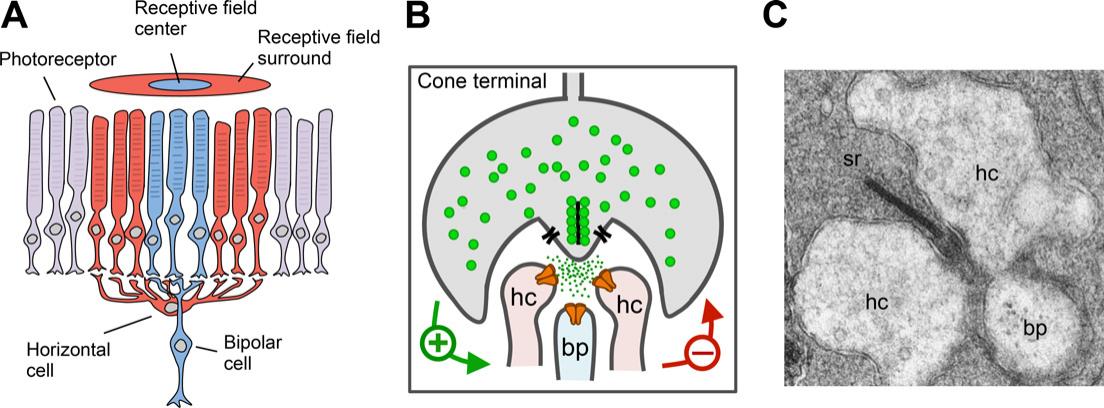
Lateral inhibition is mediated by horizontal cells (HCs) in the vertebrate retina. A. HCs collect information from photoreceptors in the receptive field surround (and center) and feed back onto photoreceptors in the receptive field center to generate the antagonistic receptive field surround of bipolar cells. B. The reciprocal synapse between cones and horizontal cells mediates negative feedback. C. Electron micrograph of the invaginating cone synapse with presynaptic ribbon (sr) and characteristic synaptic triad: lateral dendrites from two horizontal cells (hc) and a single central dendrite from a bipolar cell (bp). Panel C is from [5].

The process the scientists were studying is known as lateral inhibition, a fundamental feature of information processing in visual, mechanosensory, and auditory systems. In the visual system, lateral inhibition enhances the representation of contrast, improving perception of edges [1]. Horizontal cells (HC) are the first cellular substrates of lateral inhibition (Fig 1A) [2]. Each HC receives chemical synaptic inputs from many rods and cones, and in return, generates a feedback signal that alters their neurotransmitter release (Fig 1B). Therefore, the connection between the photoreceptors and the HCs is a reciprocal synapse. The synaptic contact between HCs and photoreceptors occurs in an invagination deep into the terminals of rods and cones, which may result in a longer path for diffusion of transmitter and electrical current out of the cleft (Fig 1B and 1C) [3]. HC feedback is crucial for establishing the antagonistic receptive field surround of the next neuron in the visual pathway (the bipolar cell), and leaves its mark on the responses of every subsequent neuron throughout the visual system [4].

## What Does the Reciprocal Synapse Do?

Lateral inhibition was first discovered in the horseshoe crab *Limulus* [6] and in cat [7], but the neural mechanism was first broached by Baylor et al. [8], who found that a large spot of light generated a voltage response in cones that was opposite in polarity to the hyperpolarizing response generated by phototransduction. The antagonistic “surround” extended for hundreds of micrometers from the center, consistent with large, laterally projecting cells, i.e., HCs. The kinetics of the surround response were similar to those of the HC light response, reinforcing this view.

Next it was found that HC feedback alters the gating of voltage-gated Ca^2+^ channels in cones. Either surround illumination or direct current injection to hyperpolarize HCs caused a shift in the cone L-type Ca^2+^ channel activation curve to more hyperpolarized potentials [9,10]. This is, therefore, a “sign-inverting” (or inhibitory) effect because HC hyperpolarization leads to cone depolarization and increased glutamate release. The sign-inverting response can be seen even in cones that have had their outer segments removed to eliminate phototransduction [11], confirming that the response is relayed by HC feedback.

Lateral inhibition and contrast enhancement are particularly important for high acuity vision in daylight, mediated mostly by cones. But recent evidence shows that HC feedback can also occur in rods [12]. In lower vertebrates, chromaticity-type HCs signal between cones that are tuned to dissimilar wavelengths of light [13]. For example, HC feedback allows red-preferring cones to inhibit green-preferring cones. This color opponency may be crucial for chromatic discrimination in these animals.

## Whodunit?

The output neurotransmitter of rods and cones was established by “sniffing” synaptic glutamate release with an excised patch containing glutamate receptors [14]. But the signal mediating HC feedback has remained at large for >50 years. Three suspects have emerged: (1) GABA, a conventional neurotransmitter; (2) protons, highly unconventional neurotransmitters; and (3) ephaptic signaling, an electrical field effect that involves no neurotransmitter. Strong arguments have been presented for and against each of these suspects, but the scientific jury is still out.

Proving that a neurotransmitter mediates a physiological response involves three types of evidence: (1) block-it results, in which a specific inhibitor of the putative mediator prevents the event; (2) move-it results, in which experimental application of the putative mediator can itself generate the event; and (3) show-it results, in which a specific sensor for the mediator in question demonstrates its presence at the right time and place, and at the right concentration. Many block-it and move-it results have been reported, but the main problem in identifying the HC feedback signal has been the relative lack of show-it results placing the mediator at the scene of the crime.

### GABA

**Fig 2:**
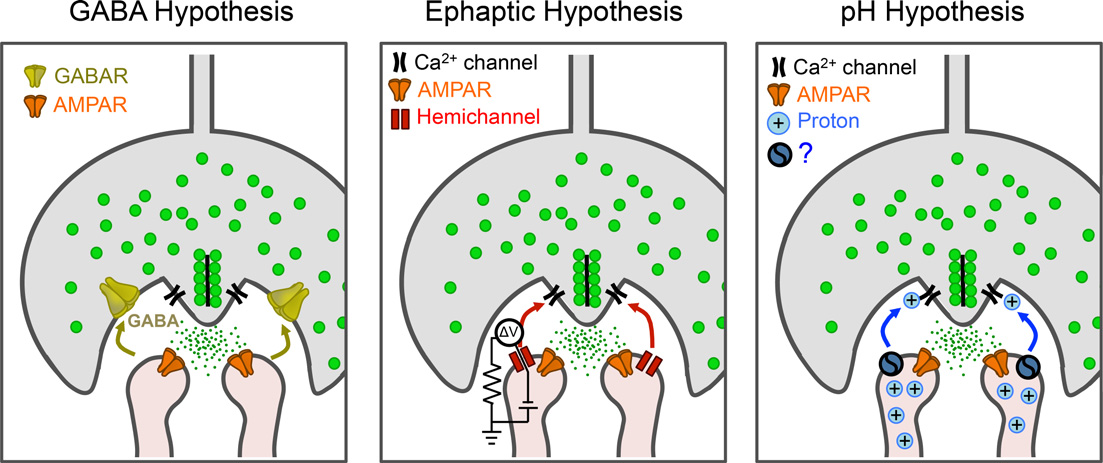
Three culprits that could mediate negative feedback from horizontal cells to cones. Cones release glutamate (green) to activate α-amino-3-hydroxy-5-methyl-4-isoxazolepropionic acid receptors (AMPAR) on HCs. HCs feed back onto cones via γ-Aminobutyric acid receptors (GABAR), an ephaptic mechanism, or synaptic pH changes.

GABA, the main inhibitory neurotransmitter in the brain, was suspected early as the HC feedback transmitter. According to the GABA hypothesis (Fig 2) glutamate released from cones in darkness depolarizes HCs, which release GABA back onto cone terminals. Activation of GABA_A_ receptors hyperpolarizes the cone terminal, suppressing activation of voltage-gated Ca^2+^ channels and reducing Ca^2+^ -dependent release of glutamate. Hence, the process is a negative feedback loop.

Early evidence supporting the GABA hypothesis in non-mammalian retina was strong. HCs express the biosynthetic enzyme for GABA, glutamic acid dehydrogenase [15], and have significant intracellular GABA [16]. GABA is released when HCs are depolarized in darkness [17]. Cone terminals have inhibitory GABA_A_ receptors [18]. Finally, the effect of surround illumination is eliminated with exogenous GABA, which would be expected to saturate the receptors [19]. More recent work in mammalian retina has identified many components of vesicular GABA-ergic transmission in HC dendrites [20].

GABA is usually released by Ca^2+^-dependent exocytosis of synaptic vesicles, but HCs fail to accumulate or release the synaptic vesicle dye FM1-43 [21]. This correlates with the HC to cone contact sites having a paucity of synaptic vesicles [22]. However, elegant studies by Eric Schwartz showed that GABA is released from HCs by an unusual mechanism in fish, amphibians, and reptiles. In these animals, GABA release is predominantly Ca^2+^ independent (though not entirely [23]) and operates through a non-vesicular pathway that involves Na^+^-coupled GABA transport [17,24]. This evidence bolstered the GABA hypothesis until it was discovered that the transporter is missing from mammals [25]. Hence, the biochemical machinery for substantial GABA release is limited to lower vertebrates.

Other results argue against the GABA hypothesis. Ultrastructural studies show few GABA receptors near the HC to cone contact sites [26]. Pharmacological studies showed that HC feedback persists after adding antagonists of all known GABA receptors (GABA_A_, GABA_B_, and GABA_c_) [10,27]. So, while the GABA hypothesis passes the move-it test, it fails the block-it test. Moreover, there is no show-it evidence for appropriately timed changes in GABA concentration with surround inhibition. The suppression of HC feedback by exogenous GABA could be explained by a decrease in the input resistance of cones and HCs, which would shunt the current generated by the natural feedback signal.

Even though the evidence appears to exonerate GABA as mediating negative feedback, the presence of some GABA_A_ receptors, mainly on HC themselves, indicates some role, perhaps as a paracrine neurotransmitter that modulates signals during light adaptation [25,28].

### Ephaptic Signal

The structure of the invaginated triad junction is reminiscent of an electronic transistor, leading to the suggestion that HC feedback might operate through a type of electrical field effect known as ephaptic transmission [29]. In the ephaptic model, current flowing through ion channels in HC dendrites encounters a high extracellular resistance, producing an extracellular voltage change. Voltage-gated Ca^2+^ channels in the cone terminal sense this change, altering their gating (Fig 2). Because extracellular depolarization is equivalent to intracellular hyperpolarization, outward current from HCs should inhibit voltage-gated Ca^2+^ channels in cones, a negative feedback effect.

To account for the proposed voltage change, the HC dendrites must possess a high density of open ion channels. The leading candidates are connexin hemichannels, which have been immunolocalized to the tips of fish HC dendrites inside the synaptic invagination [30], although not in mammals [31]. When linked between two adjacent cells, connexins form gap junctions, but some connexins form open hemichannels by themselves, providing a leakage path for current flow across the membrane of a single cell.

Both pharmacological and genetic block-it experiments have implicated connexin hemichannels in HC feedback, but none of these treatments are entirely specific for their intended target. Carbenoxolone, a gap-junction inhibitor, appears to suppress HC feedback but also directly inhibits voltage-gated Ca^2+^ channels in cone terminals [32], confounding interpretation of its effects. Moreover, paired HC-cone recordings in salamander retina show that carbenoxylone fails to block the effect of HCs on cone Ca^2+^ current [9]. HC feedback is reduced but not eliminated by a zebrafish mutation that knocks out one connexin isoform [33]. The incompleteness could be a consequence of compensatory changes in the expression of other isoforms. For these reasons, block-it experiments are inconclusive. Ionotropic glutamate receptors, also found on HC dendrites, can also suppress feedback [30]. This could constitute an alternative pathway for current flow from HCs, perhaps contributing to ephaptic signaling. However, the tighter localization of hemichannels to the portion of the HC dendrite adjacent to cone release sites may make them a more potent ephaptic conduit [34].

### Protons

Cone voltage-gated Ca^2+^ channels are acutely sensitive to extracellular pH [34,35]. Since cone Ca^2+^ channels are targets of feedback, it was suggested that HC regulation of synaptic cleft pH was the basis of lateral inhibition. According to the pH hypothesis, HC depolarization drives proton efflux through a channel or transporter in HC dendritic tips. The pH change modulates the gating of cone Ca^2+^ channels, altering Ca^2+^-dependent neurotransmitter release (Fig 2). Artificially acidifying or alkalinizing the synaptic cleft by ~0.6 log units shifts the midpoint of Ca^2+^ channel activation by ~10 mV, enough to account for HC feedback [35,36]. So, move-it evidence is consistent with the pH hypothesis.

Block-it experiments have used exogenous pH buffers to clamp the pH in the cleft. Supplementing bicarbonate, the natural buffer, with 10–20 mM HEPES, a stronger and faster buffer, suppresses HC feedback [37]. Moreover, in primate retina, HEPES eliminates the antagonistic surround of retinal ganglion cells [38], one synapse further along in the visual system.

Exogenous pH buffers may have off-target effects [39]. The aminosulfonate moiety of HEPES can directly block hemichannels. However, Tris, with no aminosulfonate, also blocks HC feedback [37]. HEPES may enter and acidify HCs [39], but other aminosulfonate buffers have no effect on HC feedback, even if they do alter intracellular pH. In fact, only those compounds with a pKa near the synaptic pH (7.4) are effective, suggesting that block is mediated specifically by pH buffering [40].

Studies on enzymatically dissociated HCs from skate retina using pH-sensitive dyes or microelectrodes show that depolarizing HCs causes extracellular alkalinization instead of the acidification predicted by the pH hypothesis [41,42]. However, these methods may not resolve hot spots of alkalinization at dendritic tips. Furthermore, enzymatic dissociation may disrupt the channels or transporters responsible for proton flux in the invaginated synapse.

Avoiding these issues are imaging experiments performed on intact zebrafish retinas expressing “CalipHluorin,” a pH-sensitive green fluorescent protein (GFP) (pHluorin) fused directly to a subunit of the cone calcium channel [43]. Changing the voltage of HCs, either with a chemical reagent that acts on an exogenously expressed ligand-gated channel, or by illuminating the surround, leads to changes in the CalipHluorin fluorescence. This indicates a change in synaptic pH, localized exactly to the site of HC feedback (the cone Ca channel). The magnitude and kinetics of the pH change match the predictions of the proton hypothesis, as determined by calibrating with solutions of known pH. According to these experiments, protons pass the show-it test as the feedback transmitter.

Still unclear, however, is the mechanism that links HC voltage to changes in synaptic pH. HCs have highly proton-permeant epithelial-type Na^+^ channels (ENaC). HC feedback is blocked by the ENaC inhibitor amiloride, but it also blocks other channel types [44]. The plasma membrane of HCs displays the vacuolar type H^+^ pump (V-ATPase). Bafilomycin-A1, a selective V-ATPase blocker, prevents proton efflux from dissociated HCs [45]. However, V-ATPase is required for vesicular glutamate uptake and bafilomycin-A1 blocks synaptic transmission, limiting its utility in the intact retina. Hemichannels are also proton-permeant, but there are no specific inhibitors, and dissociating electrical versus proton conduction is problematic.

## Towards a Solution

The 50-year mystery of HC feedback has not been solved beyond a reasonable doubt. Ideally, proof requires a combination of block-it, move-it, and show-it results, demonstrating that the putative signal is necessary, sufficient, and present at the right time and place to mediate feedback. It is important to distinguish signals that *mediate* feedback, which must meet all these criteria, from signals that *modulate* feedback, which nonetheless may play important roles in vision.

For GABA in the mammalian retina, the negative evidence far outweighs the positive. GABA agonists can inhibit cone neurotransmitter release. However, GABA receptors on the cone terminal are sparse, and there is scant evidence for vesicular release from HCs or enough GABA in the synaptic cleft. Most importantly, GABA receptor antagonists fail to block feedback.

For the ephaptic hypothesis, the case is less clear-cut. There are no specific hemichannel blockers and genetic elimination of specific connexins can trigger compensatory changes in expression of other genes. It is difficult to devise clean move-it experiments that specifically alter HC voltage, without also affecting other putative signals, such as protons. But most importantly, there is a lack of show-it results that demonstrate conclusively that voltage changes in the HCs lead to at least a local change in voltage in cones, in the complete absence of chemical communication. This criterion has been met at the basket cell–Purkinje cell synapse in the cerebellum [46], and at synapses onto the axon initial segment of Mauthner cells in the fish spinal cord [47]. In both cases, the presynaptic cell can fire action potentials, and the small signal recorded in the postsynaptic cell is transient, facilitating its measurement against slow fluctuations in background voltage. But neither HCs nor cones fire spikes, making electrophysiological measurement more challenging. However, there is another option that may be better suited for detecting local and persistent voltage changes. Several Genetically Encoded fluorescent Voltage Indicators (GEVIs) have recently been developed [48], and they can be expressed selectively in cones and targeted specifically to terminals. The usual challenge is making GEVIs that respond fast enough to detect single action potentials in individual neurons. But addressing HC feedback with GEVIs is less demanding because the signals can be spatially averaged over many cone terminals and temporally averaged over a long time period because surround responses are relatively slow and persistent.

The case seems to be strongest for the proton hypothesis, which is supported by block-it, move-it, and show-it evidence. However, the mechanism whereby HCs alter the pH of the synaptic cleft is still quite murky. Better block-it experiments are needed to establish which channel or transporter couples HC voltage to changes in proton flux. It is also possible that, rather than directly altering proton flux, feedback actually involves HC-dependent changes in the concentration of a pH buffer, such as bicarbonate [49] or phosphate [50].

Of course, it is possible that a combination of GABA, protons, and ephaptic signals coordinate to mediate HC feedback. Ephaptic transmission might contribute a fast component, while protons mediate a slower component [50], or ephaptic and proton feedback might contribute differently depending on light stimulation conditions [51]. Recent attempts to implicate a conspiracy of mechanisms rely on the same equivocal pharmacology that has muddied the roles of the individual signals. One could argue that proof of a multiplexed mechanism requires even more stringent block-it, move-it, and show-it evidence than needed to prove a unitary mechanism.

## Abbreviations

ENaC: epithelial-type Na+ channels
GABA: γ-Aminobutyric acid
GEVIs: Genetically Encoded fluorescent Voltage Indicators
GFP: green fluorescent protein
HC: horizontal cells
V-ATPase: vacuolar type H+ pump
